# Antimicrobial resistance and antimicrobial stewardship in South Africa: a survey of healthcare workers in academic and nonacademic hospitals

**DOI:** 10.1017/ash.2023.483

**Published:** 2023-11-06

**Authors:** Kessendri Reddy, Yogandree Ramsamy, Khine Swe Swe-Han, Trusha Nana, Marianne Black, Molebogeng Kolojane, Vindana Chibabhai

**Affiliations:** 1 Division of Medical Microbiology and Immunology, Department of Pathology, Faculty of Medicine and Health Sciences, Stellenbosch University, Cape Town, Western Cape, South Africa; 2 National Health Laboratory Service Tygerberg, Cape Town, Western Cape, South Africa; 3 Department of Medical Microbiology, School of Laboratory Medicine & Medical Science, College of Health Sciences, University of KwaZulu-Natal, Durban, KwaZulu-Natal, South Africa; 4 National Health Laboratory Service Inkosi Albert Luthuli Central Hospital, Durban, KwaZulu-Natal, South Africa; 5 Department of Clinical Microbiology and Infectious Diseases, School of Pathology, University of Witwatersrand, Johannesburg, Gauteng, South Africa; 6 National Health Laboratory Service Charlotte Maxeke Johannesburg Academic Hospital, Johannesburg, Gauteng, South Africa

## Abstract

**Objective:**

Antimicrobial stewardship programmes (ASPs) facilitate appropriate antimicrobial use and require contextualization for optimal functioning. We aimed to investigate perceptions of and antimicrobial resistance (AMR) and ASPs among healthcare workers in academic and nonacademic hospitals.

**Design:**

Cross-sectional survey.

**Setting:**

Three academic (Charlotte Maxeke Johannesburg Academic, Inkosi Albert Luthuli, Tygerberg) and three nonacademic hospitals (Leratong, Prince Mshiyeni Memorial, and Paarl) in South Africa from January to June 2022.

**Participants:**

Doctors, nurses, and pharmacists.

**Methods:**

Voluntary questionnaire using Google Forms, encompassing AMR, ASPs, and selected discipline-specific components.

**Results:**

Participants comprised 79 doctors (50 academic), 178 nurses (169 academic), and 21 pharmacists (18 academic) and were female predominant. AMR was a problem in academic hospitals (74.7% vs 51.2%, *p* 0.004); 73.5% overall reported inappropriate antimicrobial use as a major contributor. Adequate education on antimicrobials occurred in only 36.4% overall. Microbiological testing guided therapy more often in nonacademic settings (80.0% vs 50.2%, *p* <0.001). In both settings, antimicrobial availability drove selection in 48.2%. Overall, ASPs improved patient care (89.8%) and reduced antimicrobial use (86.9%), although felt to override prescriber autonomy in academic settings (29.4% vs 7.5%, *p* 0.007), mainly among nurses. Only 50.2% reported successful local ASPs. A minority of pharmacists (20.0%) reported sufficient hospital support for ASPs. Education, involvement of infection control staff, and inclusion of nurses in ASPs were most impactful on AMR.

**Conclusion:**

Selected healthcare worker perspectives differ by category and setting and can be targeted to improve ASPs. Further studies should target a higher number of clinical staff in both settings.

## Introduction

Antimicrobial resistance (AMR), one of the leading public health threats globally, has a significant impact in the sub-Saharan African region.^1^ The World Health Organization’s Global Action Plan on AMR lists improving awareness and understanding of AMR and strengthening the knowledge and evidence base as critical objectives.^2^ The South African National Strategy Framework on AMR aligns with this and emphasizes interdisciplinary efforts, infection prevention and control (IPC) and antimicrobial stewardship programmes (ASPs) as important enablers.^3^ However, much is dependent on behavioural change of practicing healthcare workers (HCW)^4^ and synergy between different sectors and disciplines.^5^

The delivery of healthcare in South Africa is mediated by both the public and private sectors, serving approximately 80% and 20% of the population, respectively.^6^ Academic hospitals are tertiary/quaternary facilities located within the public sector which have onsite microbiology laboratories and onsite specialist microbiologists. Nonacademic public sector hospitals receive remote microbiology laboratory and specialist support.

Understanding the differences in HCW perceptions and experiences between academic and nonacademic facilities enables effective, pragmatic targeting of interventions. A recent study on South African HCW provided critical insights at a population level.^4^ We aimed to evaluate the perceptions of AMR and ASPs among doctors, nurses and pharmacists at academic and nonacademic public sector hospitals in the three most populous South African provinces.

## Methods

### Study design and setting

Six public sector hospitals were surveyed in three provinces in South Africa. The academic facilities were Charlotte Maxeke Johannesurg Academic Hospital (CMJAH, Gauteng), Inkosi Albert Luthuli Central Hospital (IALCH, KwaZulu Natal), and Tygerberg Hospital (TBH, Western Cape). The three nonacademic facilities were Leratong Hospital (LH, Gauteng), Prince Mshiyeni Memorial Hospital (PMMH, KwaZulu Natal), and Paarl Hospital (PAH, Western Cape).

### Survey instrument and dissemination

The self-administered web-based survey, adapted from a previous survey,^7^ was distributed electronically and physically to staff members after piloting. Participation was voluntary and anonymous, and an informed consent statement was included.

The questionnaire encompassed basic demographic details, the scope of AMR and ASPs, and discipline-specific sections. Respondents also selected five interventions out of 11 options that would have the greatest impact on AMR. A free text section was offered for suggestions. Respondents could omit answers, and questions were grouped into themes for analysis.

### Statistical analysis

Data were extracted from Google Forms into Microsoft Excel 2016 (Microsoft Corporation, Washington, USA). Descriptive statistics were used (counts and proportions) to summarize the data. χ^2^ tests were used for comparison of proportions, using EpiCalc 2000 v1.02 (Brixton Health, UK). A two-tailed α of 0.05 was regarded as being statistically significant.

### Ethics

This study was approved by the University of the Witwatersrand’s Human Research Ethics Committee (reference number M200542), with the University of KwaZulu Natal’s Biomedical Research Ethics Committee and Stellenbosch University’s Health Research Ethics Committee providing reciprocal approval. Approval was granted from the provincial Departments of Health and hospital management at each site.

## Results

Two hundred and eighty-three responses were collected between January 18 and June 30, 2022. Although the sample size target was 30% of the total staff employed, the respondents represented 3.9% of the target number of doctors (3.1% in academic and 7.3% in nonacademic settings), 2.4% of the target number of nurses (2.9% in academic and 0.6% in nonacademic settings), and 12.8% of the target number of pharmacists (19.1% in academic and 4.3% in nonacademic settings). The basic demographic data of the respondents are presented as supplementary material (Supplementary Table 1); key demographics are summarized below.

From academic sites (*n* = 242), 69.8% were nurses, 20.7% doctors, and 7.4% pharmacists. The respondents were female predominant (199/242, 82.2%). TBH contributed the largest number (179/242, 74.0%), dominated by nursing staff (*n* = 151) followed by doctors (*n* = 20). CMJAH contributed the highest number of doctors (25/242, 10.3%) and pharmacists (10/242, 4.1%).

Survey responses at nonacademic sites were generally poorer (*n* = 41), mostly by doctors (70.7%). PMMH contributed 21 responses (51.2%).

### Demographics

- Doctors

Respondents were mostly specialists (*n* = 36) and medical officers (*n* = 26). The modal-age group for doctors was 31–40 years (*n* = 30, 38.0%). Most (64.6%) were female.

- Nurses

Most respondents were female (88.8%). The modal age group was 51–60 years (*n* = 53). Most reported working at their current job for 6–10 years (48/175 respondents, 27.4%).

- Pharmacists

The majority of respondents were female (66.7%). The modal age group was 21–30 years (*n* = 10). Pharmacists showed a variety of experience at their current hospitals, with seven reporting 11–20 years at their current facility (33.3%) and six reporting 0–1 year (28.6%).

### Survey responses

Survey responses by HCW category are summarized in Table 1 and below.

**Table 1.** Perceptions on antimicrobial resistance, antimicrobial stewardship, and related issues by healthcare worker category (% agreement, *n* = 278)

	Doctors (*n* = 79)	Nurses (*n* = 178)	Pharmacists (*n* = 21)	*p*-value
**Scope of antimicrobial resistance**				
AMR is a significant problem in South Africa	96.2%	83.4%	95.2%	**0.002**
AMR is a significant problem in all healthcare settings (public and private)	98.7%	79.8%	95.2%	**<0.001**
**Causes of/factors contributing to antimicrobial resistance**				
Inappropriate antimicrobial use is a major cause of AMR at the respondent’s hospital	74.7%	70.7%	95.2%	0.053
Broad-spectrum antimicrobial prescription is directly linked to AMR at the respondents’ hospital	74.7%	53.5%	66.7%	**0.008**
Interruptions of therapy contribute to AMR locally	58.2%	61.5%	85.7%	0.064
Poor infection control practices contribute to the spread of AMR locally	67.1%	55.0%	42.9%	0.072
Patients’ expectations contribute to antimicrobial overuse locally	29.1%	39.4%	61.9%	**0.020**
The local institution provides adequate staff education on antimicrobial use and AMR	41.8%	34.7%	33.3%	0.664
**Local antimicrobial resistance prevalence**				
A patient is likely to develop a hospital-acquired infection due to a drug-resistant infection during their stay	21.5%	38.3%	28.6%	0.068
**Antimicrobial stewardship programmes**				
ASPs improve quality of patient care	97.5%	85.3%	95.2%	**0.009**
ASPs reduce antimicrobial use and can save costs	97.5%	81.2%	95.2%	**0.001**
ASPs reduce duration of hospital stay	87.2%	77.9%	95.2%	0.054
ASPs reduce AMR	91.1%	76.9%	90.0%	**0.016**
ASPs impact nosocomial infection rates	89.9%	70.7%	95.2%	**<0.001**
ASPs were not needed previously so are not needed now	1.3%	18.7%	4.8%	**<0.001**
ASPs are designed to spare antimicrobial use and can negatively impact the individual patient	11.4%	38.7%	9.5%	**<0.001**
ASPs can be an obstacle to good patient care and can cause adverse outcomes	20.3%	35.7%	14.3%	**0.013**
ASPs override prescribers’ decision autonomy	18.0%	31.7%	9.5%	**0.015**
**Staff perspectives**				
Individual contributions impact AMR	88.6%	69.1%	65.0%	**0.003**
Staff do not have enough time to further invest in ASPs	7.7%	31.5%	9.5%	**<0.001**
Physicians are the only healthcare worker category needing to understand AMS	5.1%	21.4%	4.8%	**0.001**

Note. AMR, antimicrobial resistance; ASPs, antimicrobial stewardship programmes; AMS, antimicrobial stewardship.

- Scope of AMR

Staff at academic sites felt that AMR was a problem in their respective hospitals to a greater extent than staff at nonacademic hospitals (74.7% vs 51.2%, *p* 0.004), and correspondingly, 58.2% vs 41.5% reported AMR to be a problem in daily practice (*p* 0.071). Inappropriate antimicrobial use was a major contributor to AMR in 73.5% overall.

- Causes of/factors contributing to AMR

Interruptions of therapy and poor IPC practices were important contributors to AMR in 62.4% and 57.6% overall, similar in both settings. Overall, 17.0% felt that inappropriate use of laboratory diagnostic tests frequently led to overuse of antimicrobials. The lack of rapid diagnostic test availability was identified as a contributor to antimicrobial overuse in 54.4% of all respondents, higher in nonacademic settings (64.1% vs 52.8%, *p* 0.255).

- Healthcare systems

Overall, 68.4% reported adequate hospital surveillance for drug-resistant organisms. Only 36.4% felt that their hospital provided adequate education regarding antimicrobials, higher in nonacademic settings (50.0% vs 34.1%, *p* 0.079). Microbiological testing prompted appropriate changes in only 50.2% in academic settings compared with 80.0% in nonacademic settings (*p* <0.001).

Antimicrobial availability was the driving factor behind choice of agent in 48.2%, and interruptions in therapy were linked to sporadic supply/stockouts and nonadministration of prescribed agents in 42.4% and 46.8%, respectively, similar in both settings. Staff shortages were directly linked to interruptions of therapy in 31.8% overall, and the frequent lack of close clinical follow-up was identified as a contributor to AMR in 26.2%, also similar in both settings.

Overall, 80.4% of respondents felt that infectious diseases/microbiology experts were available for guidance, while only 57.1% agreed that pharmacists with sufficient training were available.

Antimicrobial stewardship (AMS) was felt to override prescriber autonomy in 29.4% of academic vs 7.5% of nonacademic respondents (*p* 0.007), driven by nurses (see Table 2).

**Table 2.** Healthcare workers’ selection of top interventions impacting on antimicrobial resistance (*n* = 283, 1415 total responses)

Interventions most frequently chosen as having the greatest impact on antimicrobial resistance	Total number of responses^a^ [*n* (%)]
Education on antimicrobial therapy to medical, pharmacy staff, and nursing staff	240 (17.0)
Active involvement of hospital infection prevention and control team	177 (12.5)
Greater involvement of nursing staff in antimicrobial stewardship	168 (11.9)
Implementation of prospective audit and feedback (multidisciplinary rounds on appropriate prescribing and use of antimicrobials)	154 (10.9)
Development of institutional guidelines for empiric antimicrobial use	143 (10.1)

a Respondents selected 5 interventions from 11 options resulting in 1415 responses.

- Antimicrobial stewardship programmes

Only 50.2% of respondents reported that their hospital had implemented an effective ASP; this was higher in nonacademic settings (57.5% vs 48.9%, *p* 0.406). In both settings, substantial support was noted for ASP improvement by the implementation of electronic medical recording (e.g. receiving results) (85.9% overall) and implementation of electronic prescribing (72.4%).

- Staffing perspectives

In academic settings, 73.0% felt that their individual contributions could impact AMR, compared with 84.6% in nonacademic settings (*p* 0.181). Feedback on antimicrobial selection was requested by 78.5% overall.

- Improvement strategies

The interventions summarized in Table 2 were selected as having the greatest impact on addressing AMR locally.

- Additional suggestions:

Comments by 45 respondents are included as supplementary material (Supplementary Table 2).

### Discipline-specific sections

- Doctors

Doctors’ responses to discipline-specific questions are summarized in Table 3. Additionally, 18.0% used broad-spectrum agents (e.g., meropenem and vancomycin) for very sick patients regardless of risk of hospital-acquired infection, information on which was only sought in 74.0% overall.

**Table 3.** Doctors’ responses to discipline-specific questions by setting (*n* = 79)

	Academic	Nonacademic	*p*-value
	(*n* = 50; % of respondents)	(*n* = 29; % of respondents)	
Microbiology results are available timeously	71.4	55.2	0.225
I routinely check microbiology results to guide therapy	98.0	93.1	0.639
Updated local antibiograms were available in the last year	30.6	41.4	0.472
I routinely de-escalate from intravenous to oral therapy at around Day 3, if clinically possible	91.8	86.2	0.685
I routinely de-escalate from broad-spectrum to narrow-spectrum agents	95.9	79.3	0.051
I routinely assess the need for continued antimicrobials	93.9	89.7	0.813
I obtain information on previous hospitalization or antimicrobial exposure in all patients	71.4	75.9	0.871
Restriction policies impair provision of good patient care	20.4	31.0	0.433

- Nurses

**Figure 1.**
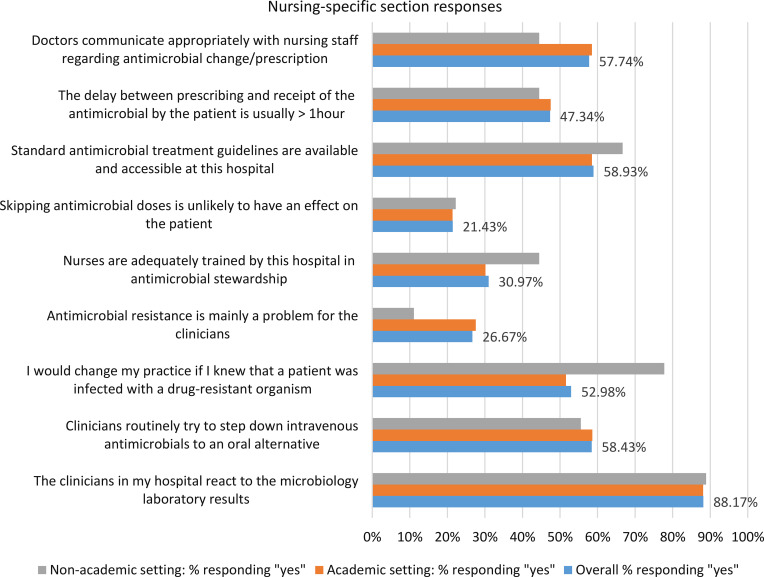
Responses to the nursing-specific section of the survey, by academic and nonacademic setting (*n* = 178, % in agreement)

Nursing-specific responses are summarised in Figure 1. No statistically significant differences were noted.

- Pharmacists

**Figure 2.**
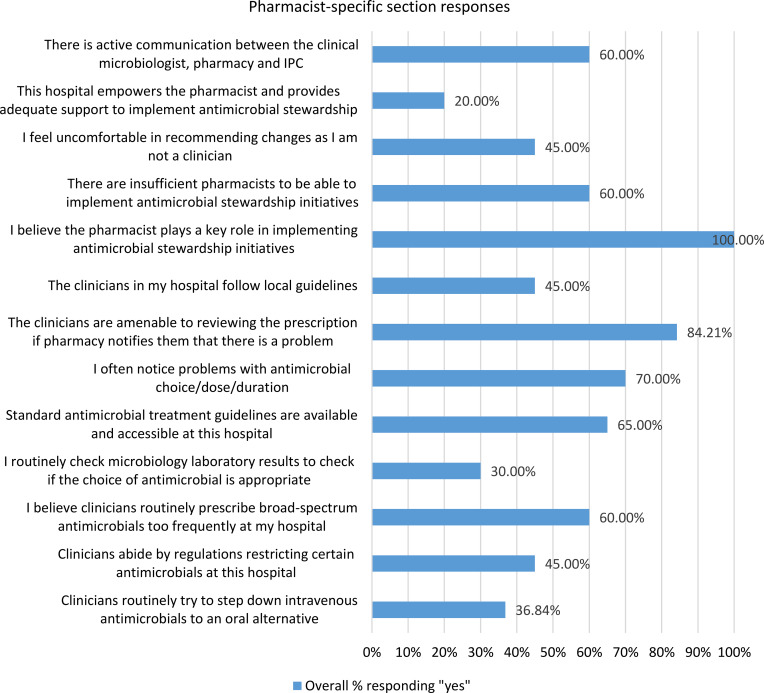
Responses to the pharmacist-specific section of the survey (*n* = 21, % in agreement)

Responses from 21 pharmacists are summarized in Figure 2 in totality. Although a substantial majority of doctors reported routinely switching from intravenous to oral antimicrobial formulations, only 36.8% of pharmacists concurred.

## Discussion

This is the first study assessing HCW perceptions in academic compared with nonacademic public sector hospitals in South Africa and supplements the evidence on AMR and ASP insight locally and globally, including in the One Health context.^4,8–15,18–23^ The study surveyed a range of ages and work experience in three provinces. Several notable differences were found between academic and nonacademic environments necessitating contextualized approaches.

AMR is widely perceived by HCW as a serious problem globally and nationally,^4,7–9^ with fewer nurses in agreement.^4,18^ This study also showed that a lower proportion of HCW considered AMR a problem at their facility, pronounced in nonacademic settings, in keeping with other findings.^4,7–9,14,15,20–23^ Adequate education on antimicrobials, reported by 36.4% of respondents in this survey, remains crucial. More than one-quarter of doctors and nurses felt that inappropriate antimicrobial use was not linked to AMR and 42.4% overall (mostly nurses and pharmacists) did not agree that poor IPC practices contributed to AMR, a finding that has been noted amongst South African medical students and physicians and pharmacists in Ethiopia.^7,8^ Although national IPC recommendations are in place, successful implementation of these seemingly low-hanging fruit remains a challenge in overburdened and poorly resourced healthcare facilities.^23,24^ This cognitive dissonance is striking given that approximately one-third of respondents at academic facilities, mostly nurses, felt it very likely that a patient would develop an infection with a drug-resistant organism during their hospital stay similar to a study in Indonesia and lower than the 66.0% in Ethiopia.^7,13^ Only 53.0% of nurses overall would change their practice if a patient were known to be infected with a drug-resistant organism.

The tendency to underestimate the impact of individual actions in achieving a greater goal is a logical fallacy. This study showed that a significantly higher proportion of doctors felt that their individual contributions could impact AMR, emphasizing the need to empower nurses and pharmacists to counteract externalization of responsibility.^12^ Education and active involvement of the hospital IPC team were among the most frequently selected recommendations thought to have the greatest impact on tackling AMR, suggesting a lack of accountability or suboptimal knowledge on the importance of IPC. A comprehensive approach to understanding behavioral aspects and cognitive biases in AMR and AMS is needed.^25^

Diagnostic stewardship, including implementation of rapid diagnostic tests, can provide supplementary or quicker decision support for antimicrobial prescription and can guide microbiology result interpretation and inform appropriate collection of samples.^26^ This is highly relevant in both settings; in the academic setting, sample collection changed therapy in just 50.2% of patients, contributing to unnecessary resource consumption. In the nonacademic setting, the lack of availability of rapid diagnostic tests contributed to antimicrobial overuse in 64.1%, similar to the 64.4% of respondents at a tertiary center in Ethiopia but higher than the 52.8% reported in academic settings.^7^ Microbiology laboratories are traditionally located in academic settings outlining the need to optimize communication between the laboratory and clinicians, as highlighted previously.^7,13^

A greater proportion of staff in academic facilities, especially nurses, felt that ASPs override prescriber autonomy and can negatively affect patient care. These findings may be explained by the presence of more experienced personnel in South African academic settings and underscores the need for synergism between different professionals and education on the goals of AMS.^15,21^ Previous reports from Thailand and Indonesia found that doctors, rather than nurses or pharmacists, objected to interventions limiting prescribing decisions, while only 21.3% of physicians in Ethiopia reported that ASPs impacted decision autonomy.^7,15,21^

Exposure to effective ASPs was only reported by 50.2% of respondents in this study. Almost a third of nurses in academic settings did not have time to further invest in ASPs and felt that only clinicians needed to understand AMS; this may be reflective of different professional demands on their time or an alternative perspective on the role of nurses in successful ASPs.^15^ Despite this, greater involvement of nursing staff in ASPs was selected among the top five interventions having an impact on AMR with adequate training reported by 31.0% of nursing staff overall. Significantly fewer nurses felt that ASPs reduce antimicrobial use and can save costs, improve quality of care, reduce AMR and duration of hospital stay, impact nosocomial infection rates, and are currently needed. The important role of nurses in AMS has been recognized, with education, optimization of interprofessional dynamics, and support of nursing leadership being strategic enablers.^15,17^

Access to essential medicines of assured quality is a key component in optimizing antimicrobial use.^2^ When indicated, it is imperative that the appropriate antimicrobials are accessible. Almost half of this survey’s respondents reported that availability drove selection compared with 68.9% in Ethiopia, 63.8% in the Democratic Republic of Congo, and 14.0% in Peru.^7,11,20^ Timely administration is also fundamental and reduces mortality in patients with sepsis;^27^ almost half of the nursing staff reported interruptions in therapy due to nonadministration of prescribed agents and delays between prescription and administration of longer than one hour. Staff shortages may play a role; utilization of technology to implement more rapid delivery of results or electronic prescribing garnered substantial support in this survey and may ease the effects of staffing challenges.

Although the overwhelming majority of doctors reported routinely assessing for de-escalation and switching to oral formulations, in agreement with data from junior doctors in France, this was not corroborated by nursing staff nor pharmacists.^19^ Possible reasons include recall or performance bias or the fact that pharmacists and nurses have exposure to prescriptions written by a variety of doctors including those who were not survey participants. Prescription practices should be targeted as a quality improvement initiative as identified by doctors and nurses in Gabon, as 70.0% of pharmacists in this study often noted errors in antimicrobial prescriptions.^10^ Poor adherence to antimicrobial prescription guidelines (45.0%) has been previously reported in South Africa.^28^

Additional pharmacists, infectious disease physicians, or clinical microbiologists were not viewed as having the greatest impact on AMR, contrary to previous studies.^8,10,14,30^ These skilled personnel are, however, essential in enabling some of the interventions chosen as most impactful in the present and previous studies, such as education, multidisciplinary rounds (with prospective audit and feedback), and institutional guideline development.^7,13,15,18,30^ Pharmacists approach antimicrobial therapy from a systems perspective and are integral to successful ASPs;^6,7,15,21,29^ in this survey, 42.9% of all respondents did not feel that sufficiently trained pharmacists were available. The vast majority of pharmacists (80.0%) felt that they were not supported by their hospital to implement ASPs, while over 60% reported patient expectations as a contributor to antimicrobial overuse. The vital role of financial, information technology, and management support in effective ASPs was echoed in this survey.^29^

Limitations to this study include volunteer bias, which is difficult to mitigate in this type of research, and survey availability only in English. Lack of dedicated time, the absence of incentives, and restrictions in mobile data may have influenced participation; paper-based surveys were also distributed on request. The length of the survey may have dissuaded potential participants. There was a substantial female predominance; it is unclear whether this represents the total population of HCW. The modal age groups differed notably between professional groups. Generational differences, varying durations of working experience, and differing training/knowledge (reflective of undergraduate and postgraduate curriculum trends) may have influenced the responses as previously reported.^13,14,17,18,31^ The small sample size overall prohibited firm conclusions with no category reaching the desired participant number; while poor response rates are a recognized limitation of surveys, stronger support from hospital management and unit managers may have improved participation and facilitated collection of more representative data.

Perceptions on AMR and ASPs differed somewhat between academic and nonacademic settings in this multicenter survey although many similarities were noted. AMR was of more concern in academic settings. In nonacademic settings, staff were willing to allocate more time to ASPs. Diagnostic stewardship, prescribing practices, and antimicrobial access must be optimized in both settings. IPC practices are underestimated as a contributor to AMR. Education, involvement of the hospital IPC team, and inclusion of nursing staff in ASPs were chosen as having the greatest impact on AMR. A multidisciplinary behavioral science-driven approach is needed to address the complex issue of AMS among HCW.

## Supporting information

Reddy et al. supplementary material Reddy et al. supplementary material

## Supplementary material

For supplementary material accompanying this paper visit http://doi.org/10.1017/ash.2023.483. click here to view supplementary material

## Research transparency and reproducibility

The relevant anonymized data collected in this study will be made available upon reasonable request.

## Previous presentation

Preliminary data on a minority of these findings were presented as a poster presentation at a local congress (FIDSSA 2022).
