# Supplementary material for: Antimicrobial resistance and antimicrobial stewardship in South Africa: a survey of healthcare workers in academic and nonacademic hospitals
Source: Antimicrob Steward Healthc Epidemiol. 2023 Nov 6;3(1):e202. doi: 10.1017/ash.2023.483 (PMC10654946; doi:10.1017/ash.2023.483)
Supplement: Reddy et al. supplementary material [file S2732494X23004837sup001.docx]

# Supplementary Material

**Supplementary Table 1:** **Demographic characteristics of the healthcare workers participating in the survey**

|  | **Academic (n=242)** | | | | **Non-academic (n=41)** | | | |
| --- | --- | --- | --- | --- | --- | --- | --- | --- |
|  | **Charlotte Maxeke Johannesburg Academic Hospital (n=50)** | **Inkosi Albert Luthuli Central Hospital (n=13)** | **Tygerberg Hospital (n=179)** |  | **Leratong Hospital (n=11)** | **Prince Mshiyeni Memorial Hospital (n=21)** | **Paarl Hospital (n=9)** |  |
| Gender | | | | | | | | |
| Female | 36 | 12 | 151 | **199** | 7 | 14 | 8 | **29** |
| Male | 14 | 1 | 20 | **35** | 4 | 6 | 1 | **11** |
| Prefer not to say/blank | 0 | 0 | 8 | **8** | 0 | 1 | 0 | **1** |
| Age | | | | | | | | |
| 21-30 years | 12 | 1 | 23 | **36** | 7 | 1 | 3 | **11** |
| 31-40 years | 12 | 4 | 57 | **73** | 1 | 8 | 3 | **12** |
| 41-50 years | 11 | 4 | 47 | **62** | 2 | 10 | 3 | **15** |
| 51-60 years | 11 | 4 | 48 | **63** | 1 | 2 | 0 | **3** |
| > 60 years | 4 | 0 | 4 | **8** | 0 | 0 | 0 | **0** |
| Years of service at current hospital | | | | | | | | |
| 0-1 year | 10 | 0 | 18 | **28** | 7 | 1 | 3 | **11** |
| 2-5 years | 12 | 3 | 40 | **55** | 1 | 5 | 4 | **10** |
| 6-10 years | 7 | 4 | 50 | **61** | 1 | 7 | 0 | **8** |
| 11-20 years | 10 | 6 | 33 | **49** | 2 | 5 | 2 | **9** |
| 21-30 years | 6 | 0 | 16 | **22** | 0 | 2 | 0 | **2** |
| > 30 years | 3 | 0 | 22 | **25** | 0 | 0 | 0 | **0** |
| Blank | 2 | 0 | 0 | **2** | 0 | 0 | 0 | **0** |
| Position at hospital | | | | | | | | |
| Doctor | 25 | 5 | 20 | **50** | 9 | 12 | 8 | **29** |
| Nurse | 12 | 6 | 151 | **169** | 1 | 7 | 1 | **9** |
| Pharmacist | 10 | 1 | 7 | **18** | 1 | 2 | 0 | **3** |
| Other* | 3 | 1 | 1 | **5** | 0 | 0 | 0 | **0** |

* Other: Clinical programme coordinator (TBH), laboratory technologist/technician (IALCH), Manager (CMJAH), Infection Control Coordinator (CMJAH), Clinical education and training manager (CMJAH)

**Supplementary Table 2: Feedback given by healthcare workers in the open text component of the survey (n = 45)***

| **Theme** | **Responses (n, %) (n = 66)*** |
| --- | --- |
| Skills/training-related, e.g. multidisciplinary rounds, guideline compliance, in-service training | 22 (33.3%) |
| Personnel-related, e.g. more microbiologists (at least one per hospital), lack of consultant availability in the wards to cosign for antibiotics, more cleaning staff/household aids | 14 (21.2%) |
| Resource-related, e.g. ward space, empiric guideline availability, time | 8 (12.1%) |
| Pharmacy-related, e.g. authorised prescribers to be identified who can be contacted regarding resistance patterns, more awareness of contributions for clinical pharmacists, pharmacists to do ward rounds | 7 (10.6%) |
| Infection-control related, e.g. proper cleaning of wards, more guidance from IPC sisters, spread of organisms in the ward of concern | 6 (9.1%) |
| Information technology-related, e.g. computerized cumulative reports for individual patients, regular antibiograms and antibiotic use data, monitoring of prescriptions | 5 (7.6%) |
| Laboratory testing-related, e.g. antibiotic drug levels, prompt phoning out of positive blood cultures, waiting for result before starting antimicrobials | 4 (6.1%) |

*More than one response extracted from the comments so total exceeds the respondent denominator of 45
